# MGMT promoter methylation modulates the effect of residual tumor on survival after re-resection of recurrent glioblastoma

**DOI:** 10.1186/s40478-026-02234-w

**Published:** 2026-02-12

**Authors:** Obada T. Alhalabi, Lukas Klein, Kirill Mironov, Lukas Beyl, Tobias Kessler, Christine Jungk, Philipp Sievers, Felix Sahm, Martin Bendszus, Wolfgang Wick, Bogdana Suchorska, Sandro M. Krieg, Sebastian Ille

**Affiliations:** 1 Department of Neurosurgery, Heidelberg University Hospital, Ruprecht-Karls- University Heidelberg, Im Neuenheimer Feld 400, 69120 Heidelberg, Germany; 2 Clinical Cooperation Unit Neurooncology, German Cancer Research Center (DKFZ), Heidelberg, Germany; 3 Department of Neurology and Neurooncology Program, National Center for Tumor Diseases, University Hospital Heidelberg, Heidelberg, Germany; 4 Department of Neuropathology, Institute of Pathology, Heidelberg University Hospital, Heidelberg, Germany; 5 Clinical Cooperation Unit Neuropathology, German Consortium for Translational Cancer Research (DKTK), German Cancer Research Center (DKFZ), Heidelberg, Germany; 6 Department of Neuroradiology, University Hospital Heidelberg, Heidelberg, Germany

**Keywords:** Recurrent glioblastoma, Intraoperative magnetic resonance imaging, Volumetric analysis, Neurological morbidity

## Abstract

Surgical re-resection for recurrent IDH–wildtype glioblastoma remains controversial, and no molecular biomarkers currently inform on tailoring the extent of cytoreduction and hence onco-functional balance at recurrence. On the other hand, while the prognostic relevance of O6-methylguanine-DNA-methyltransferase (MGMT) promoter methylation is established in glioblastoma. Its role in surgical decision-making at recurrence remains unclear. We analyzed a cohort of 153 patients who underwent re-resection for IDH-wildtype recurrent glioblastoma (WHO classification 2021) between 2015 and 2024. Volumetric analysis of contrast-enhancing (CE) tumor on pre- and early postoperative MRI was performed. Patients were stratified by MGMT promoter methylation status and residual tumor volume (RTV): 0 ml, < 1 ml, > 1 ml. Functional outcomes up to one year after surgery were longitudinally assessed using the National Institutes of Health Stroke Scale (NIHSS). There was no significant difference in RTV between MGMT-methylated (*n* = 58, 38%) and unmethylated patients (*p* > 0.999). The benefit from complete or near-complete resection was more pronounced in MGMT-unmethylated tumors, with a median postoperative survival of 288 days for 0 to 1 ml RTV versus 190 days > 1 ml (*p* = 0.024; difference = 98 days), compared to MGMT-methylated tumors with a median survival of 411 days versus 378 days (*p* = 0.043; difference = 33 days). MGMT-unmethylated patients with 0 ml RTV showed significantly higher KPS values six weeks postoperatively (*p* < 0.05) compared to higher RTVs. Postoperative deficits were comparable across groups. These findings identify MGMT promoter methylation as a clinically actionable molecular modifier of the oncological benefit of cytoreduction and support MGMT-stratified, precision neurosurgical strategies for recurrent glioblastoma, especially around functionally eloquent tumors.

**Supplementary Information:**

The online version contains supplementary material available at 10.1186/s40478-026-02234-w.

## Introduction

Glioblastoma (glioblastoma) is the most common primary malignant brain tumor in adults and remains uniformly lethal despite maximal-safe resection followed by radiotherapy and temozolomide chemotherapy [31, 32, 38, 39]. Almost all tumors recur with no universally established standard‐of‐care at first relapse [3, 20]. Therapeutic strategies vary widely among centers, and only about one third of patients are considered for re-resection, depending on tumor location, neurological status, and anticipated morbidity [35, 43].

Re-resection has been linked to prolonged survival in several large retrospective series [28, 33], particularly for patients with non-eloquent but symptomatic tumors with a pre-operative Karnofsky performance scale (KPS) > 60 [2, 25, 42]. These studies showed satisfactory functional data in affected patients, with rates of permanent new post-operative deficits after re-resection of recurrent glioblastoma at 8–16% [1, 24, 28]. In addition, the magnitude of benefit appears to depend strongly on the extent of resection (EOR) and, more precisely, on the residual contrast-enhancing tumor volume (CE-RTV) [11, 22]. According to the Response Assessment in Neuro-Oncology (RANO) resect group, complete or near-complete cytoreduction (RANO classes 1 and 2) is associated with improved survival. At the same time, additional removal of non-contrast-enhancing (non-CE) FLAIR-hyperintense tissue confers no further survival benefit in patients with recurrent glioblastoma [17].

Epigenetic silencing via methylation of the DNA repair enzyme O⁶-methyl-guanine-DNA-methyl-transferase (*MGMT*) promoter has emerged as a strong prognostic biomarker that is predictive of responsiveness to alkylating agents in IDH-wildtype glioblastoma [12, 13, 40]. Previous studies have reported on the importance of the *MGMT* promoter status in the context of neurosurgical resection strategies for glioblastoma [10, 15]. Indeed, subgroup analyses showed that achieving 0 ml residual contrast-enhancing tumor volume (CE-RTV) was associated with a greater prolongation of overall survival (OS) in patients with an unmethylated *MGMT* promoter compared to those with a methylated promoter in primary glioblastoma [29]. Whether this effect also holds in the recurrent setting has not been systematically examined thus far.

In this study, we examine the prognostic impact of CE-RTV on patient survival following re-resection of recurrent glioblastoma, depending on *MGMT* promoter methylation status, and contextualize this within the notion of onco-functional balance by including post-operative and follow-up data on neurological morbidity and quality of life of affected patients.

## Methods

### Study population

A consecutive cohort of patients with recurrence of a previously resected IDH wildtype glioblastoma (as per WHO 2021 classification) was retrospectively identified from our institutional database who underwent re-resection between 2015 and 2024 at a tertiary neuro-oncology center. Pre-operative MRI (preopMRI, 3 Tesla) and early post-operative MRI (epMRI, 3 Tesla, within 48 h), including contrast-enhanced T1-weighted sequences, were analyzed for tumor volumes. Demographic characteristics (such as age and sex) and clinical information including prior treatments (surgery, radiotherapy, chemotherapy), tumor location, neurological status (NIHSS, seizure history), and postoperative outcomes were extracted from the medical charts of Heidelberg University Hospital.

### MGMT promoter status

O6-methylguanine-DNA methyltransferase (MGMT) promoter status was determined through pyrosequencing or through methylation arrays, both at primary tumor occurrence and/or recurrence. For pyrosequencing, a mean methylation level ≥ 8% across CpG sites was used as the cutoff to define MGMT promoter methylation, consistent with established clinical practice at our institution and in prior studies.

### Tumor volumetry

Volumetric analysis of pre-operative, intraoperative, and early post-operative MRI scans was performed using the Brainlab™ software SmartBrush (Brainlab, Germany) for measurement of the absolute tumor volume of the CE tumor regions. The Response Assessment in Neuro-Oncology (RANO) Resect group classification criteria were utilized to determine residual contrast-enhancing tumor.

### Study endpoints

Patients were followed until death or loss to follow-up (censored on the day of last follow-up), with survival after repeat surgery defined as the interval from the date of repeat surgery to death from any cause or censorship. Transient post-operative neurological deficits were defined as deficits resolving within 30 days; those persisting beyond this period were classified as permanent.

### Statistical analysis

Patient characteristics were initially plotted using descriptive statistics. We report continuous variables reported as means ± standard deviation and/or median (and interquartile range (IQR)). Ordinal and nominal variables are presented as numbers and percentages. Missing data are designated as such. Comparison of nominal variables between groups was performed using the Chi-Square and Fisher’s exact test. Survival analysis was performed using the Log-rank (Mantel-Cox) test. All statistical analyses were performed using Graphpad PRISM (Version 10) and the SPSS 23 software by IBM.

## Results

### The heterogeneous landscape of surgically treated patients with recurrent glioblastoma

**Fig. 1:**
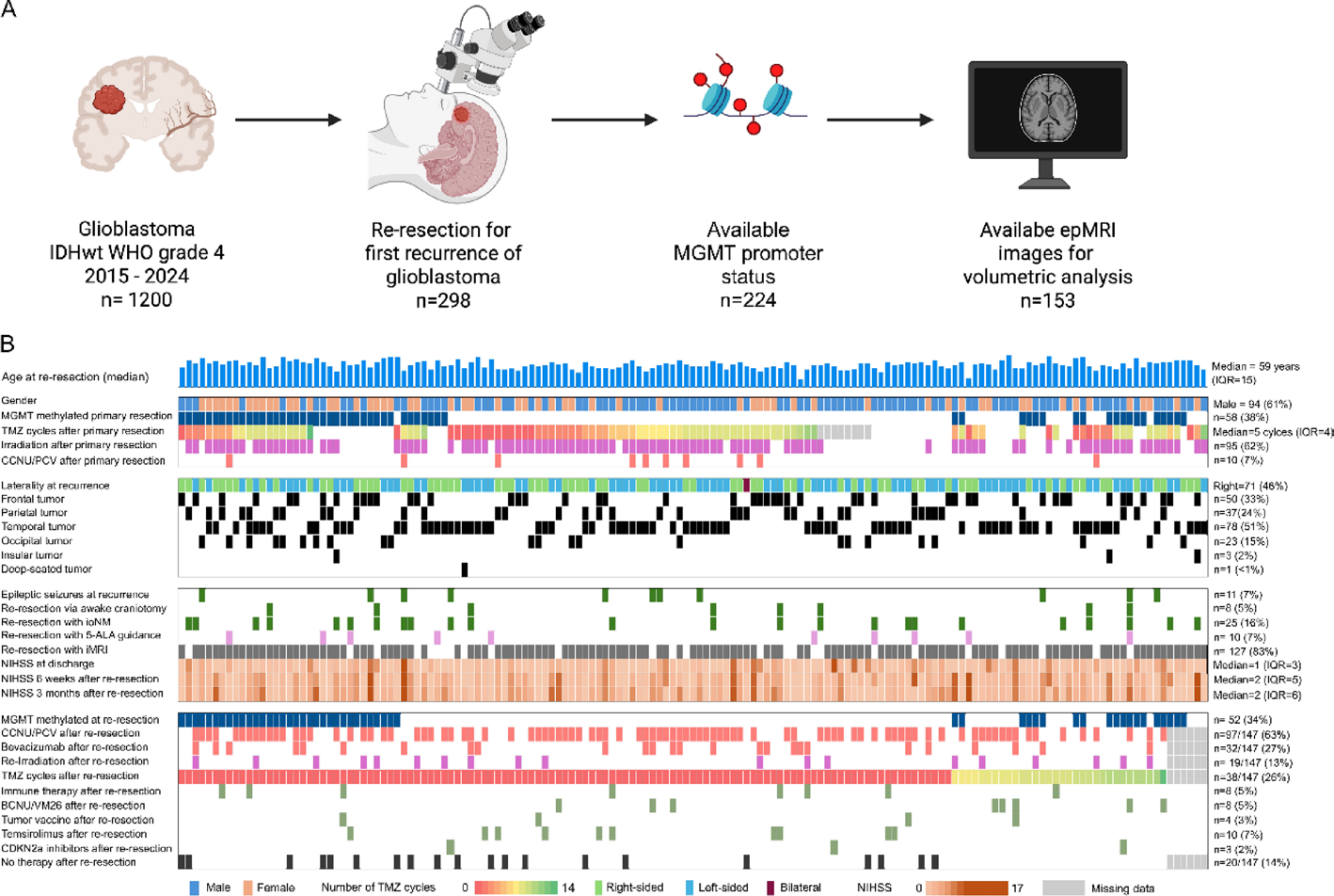
The heterogeneity of treatment recurrence in glioblastoma patients after re-resection. **A**: Study flowchart. IDH Isocitrate dehydrogenase. *MGMT* = O⁶-methyl-guanine-DNA-methyl-transferase. epMRI: early post-operative MRI. **B**: Oncoplot of *n* = 153 patients included in this study (across). TMZ = Temozolomide. Io mapping = intraoperative neuromonitoring. 5-ALA = 5-aminolevulinic acid. iMRI: intraoperative MRI; NIHSS: National Institutes of Health Stroke Scale; CCNU: Lomustine; VP-16: Etoposide; BCNU: Carmustine; VM-26: Teniposide

Between 2015 and 2024, a total of 1200 patients with histologically confirmed, IDH-wildtype (IDHwt), WHO grade 4 glioblastoma were surgically treated at our institution. Of these, 298 patients underwent surgical re-resection at first recurrence (Fig. 1A). MGMT promoter methylation status from the time of initial diagnosis was available for 224 patients, comprising the molecularly annotated cohort. Additionally, epMRI imaging suitable for volumetric assessment was available in 153 patients, forming the final analysis cohort of this study. Median age at re-resection was 59 years (IQR, interquartile range 15); 61% were male. At primary diagnosis, 58 patients harbored a methylated *MGMT* promoter (38%), while the remaining 95 patients (62%) were unmethylated. Following initial surgery, most patients received temozolomide (TMZ) chemotherapy, with a median of 6 cycles (IQR = 4). Irradiation was utilized in 62%, while CCNU/lomustine was used in 7%. At the time of recurrence, tumors were most frequently located in the temporal (51%) and frontal (24%) lobes. Right-sided tumors were slightly less represented (46%). Deep-seated tumors were observed in only 2% of the cases. The pre-operative NIHSS of re-resected patients was 1 (interquartile range IQR = 3). Epileptic seizures at recurrence were reported in 11% of patients, with a median score of 1 at discharge (IQR, 3), two at 6 weeks (IQR, 5), and one at 3 months (IQR, 6) following re-resection. Re-resections were commonly performed under the resection-guidance of intraoperative MRI (iMRI, 83%), with 5-ALA (5-aminolevulinic acid) fluorescence guidance used in only 7% of the cases. Awake craniotomy was employed in 5% of the cases, with intraoperative neuromonitoring in 16%. Post-re-resection therapy was heterogeneous. The most administered regimens were PCV (procarbazine, lomustine [CCNU], vincristine) or CCNU/lomustine only chemotherapy (63%) and/or temozolomide rechallenge (26%), bevacizumab (27%), and re-irradiation (13%). Investigational or targeted therapies included immune checkpoint inhibitors or BCNU/VM26 (5% each), tumor vaccines (3%), temsirolimus (7%), and CDK4/6 inhibitors (2%). Other treatments, including BRAF/MEK inhibitors and experimental agents, were used less frequently (< 2%). In 14% of the patients, no therapy was applied (Fig. 1B; Table 1).

**Table 1:** Patient characteristics of the final study cohort

Patient characteristics	n=153 (%)
Gender	
Male	94 (61)
Female	59 (39)
*MGMT* Status	
Methylated	58 (38)
Unmethylated	95 (62)
Age	
Mean (SD)	58.6 (11)
Median (IQR)	59.0 (15)
Karnofsky at baseline	
Median (IQR)	90 (10)
Hemisphere	
Right hemisphere	71 (46)
Left hemisphere	82 (55)
Lobe involvement	
Frontal	50 (33)
Parietal	37 (24)
Temporal	78 (51)
Occipital	23 (15)
Insula	3 (2.0)
Deep	1 (<1)
Treatment	
Lomustine/CCNU	91 (60)
Bevacizumab	26 (17)
Temozolomide	32 (21)
Study medication^a^	11 (7)
No adjuvant treatment	20 (13)
Re-irradiation	19 (12)

^a^Chi-Square-Test. One-way Analysis of Variance (ANOVA). PCV Procarbazine, lomustine (CCNU), and vincristine. Other agents include carboplatin and hydroxycarbamide abemaciclib. SD Standard deviation. IQR Inter-quartile Range. *MGMT* Status O6-methylguanine-DNA methyltransferase promoter status

**Fig. 2:**
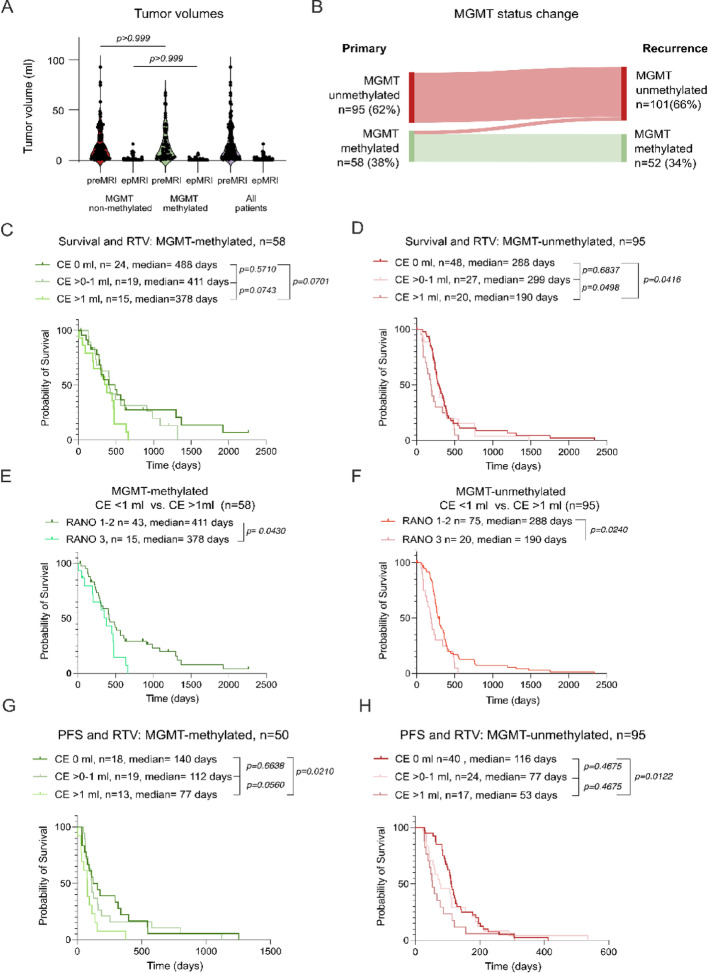
Utility of *MGMT* promoter status for tailoring resection in recurrent glioblastoma. **A**: Violin plots of tumor volumes on preoperative MRI (preopMRI) and early postoperative MRI (epMRI) in patients with *MGMT*-methylated (*n* = 58) and unmethylated tumors (*n* = 95). Tukey’s multiple comparisons test. **B**: Sankey diagram illustrating *MGMT* promoter methylation status at primary diagnosis and at recurrence. **C**: Kaplan–Meier survival analysis in *MGMT*-methylated tumors stratified by residual tumor volume (0 ml, > 0–1 ml, > 1 ml). Log-rank (Mantel-Cox) test with p-values embedded in the Figure. **D**: Kaplan–Meier survival analysis in *MGMT*-unmethylated tumors stratified by residual tumor volume (CE-RTV), demonstrating significantly shorter survival in patients with CE-RTV > 1 ml (*p* = 0.0416). **E**: Kaplan–Meier survival analysis comparing patients with CE-RTV ≤ 1 ml (RANO class 1–2) vs. > 1 ml (RANO class 3) in *MGMT*-methylated tumors, Log-rank (Mantel-Cox). **F**: Kaplan–Meier survival analysis comparing CE-RTV ≤ 1 ml vs. > 1 ml in *MGMT*-unmethylated tumors, showing a pronounced survival difference (median survival 286 vs. 190 days; *p* = 0.0240, Log-rank (Mantel-Cox)). **G**: Progression-free survival stratified by CE-RTV in *MGMT*-methylated patients, with significantly shorter PFS in patients with CE-RTV > 1 ml, Log-rank (Mantel-Cox). Progression-free survival stratified by CE-RTV in *MGMT*-unmethylated patients, demonstrating shorter PFS with increasing residual tumor burden

**Fig. 3:**
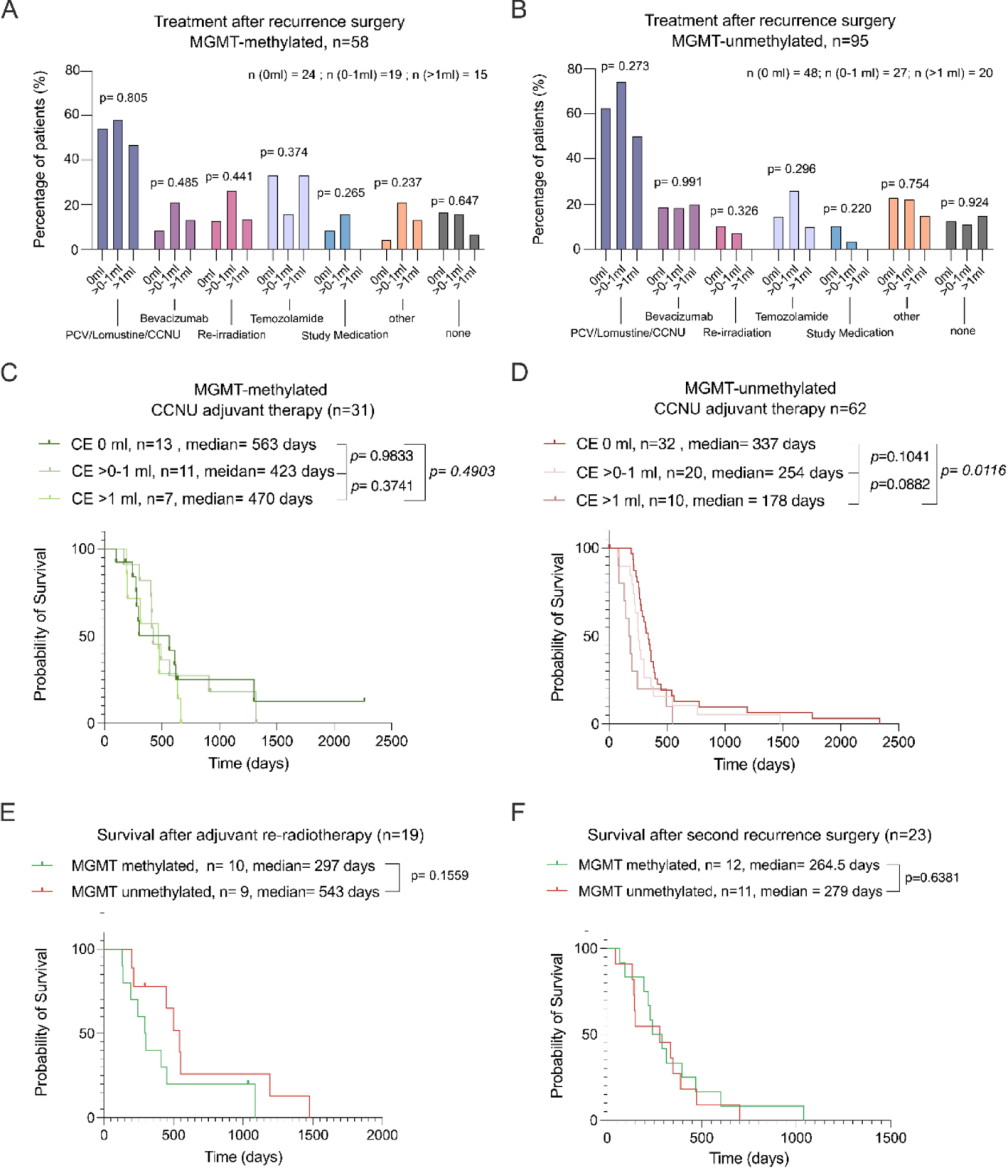
Treatment after re-resection surgery for recurrent glioblastoma. **A**: Distribution of post-operative treatments in *MGMT*-methylated patients stratified by CE-RTV class. No significant differences in treatment allocation were observed (Chi-Square-test). **B**. Distribution of post-operative treatments in *MGMT*-unmethylated patients stratified by CE-RTV class. **C**: CE-RTV class-stratified survival after re-resection in *MGMT*-methylated patients receiving adjuvant CCNU-based chemotherapy, stratified by CE-RTV, Log-rank (Mantel-Cox) test. **D**: CE-RTV class-stratified survival after re-resection in *MGMT*-unmethylated patients receiving adjuvant CCNU-based chemotherapy, stratified by CE-RTV, Log-rank (Mantel-Cox). **E**: Survival after re-irradiation stratified by *MGMT* methylation status, showing no statistically significant difference, Log-rank (Mantel-Cox) test. **F**: Survival after re-resection at second recurrence ‘re-re-resection’ stratified by *MGMT* methylation status, showing no statistically significant difference, Log-rank (Mantel-Cox) test

**Fig. 4:**
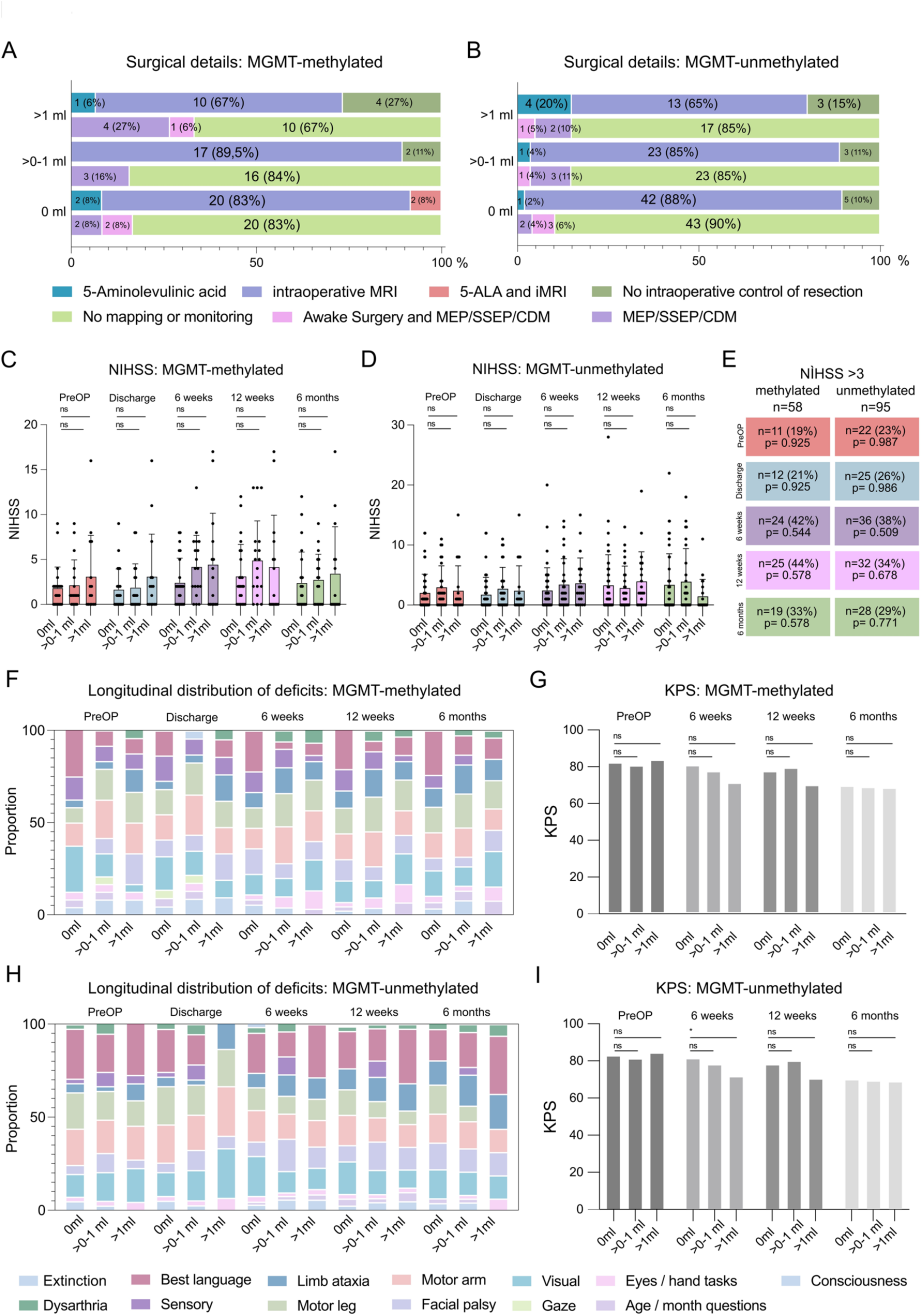
Minimizing residual tumor volume does not compromise functional integrity in recurrent glioblastoma. **A**: Distribution of intraoperative mapping, monitoring, and resection control techniques by residual tumor volume in patients with *MGMT-*methylated tumors and **B**: MGMT-unmethylated tumors. The proportions of patients receiving different combinations of 5-aminolevulinic acid (5-ALA), intraoperative MRI (IoMRI), awake mapping, and monitoring modalities are shown. They are not significantly overrepresented in any of the groups or by residual tumor volume (CE-RTV) classes. Chi-Square test. Io mapping = intraoperative neuromonitoring. 5-ALA = 5-aminolevulinic acid. iMRI intraoperative MRI. **C**: NIH Stroke Scale (NIHSS) scores over time stratified by residual tumor volume in MGMT-methylated tumors and **D**: MGMT unmethylated tumors. Boxplots show median, interquartile range, and outliers at preoperative, discharge, 8 weeks, 12 weeks, and 6-month timepoints, Tukey’s multiple comparisons test. **E**: Proportion of patients with NIHSS > 3 at 6 months postoperatively, stratified by MGMT status and residual tumor volume, with *p*-values corresponding to the probability of an overrepresentation of the event of relevant neurological deficits (NIHSS > 3) within a given group and time point, Chi-Square test. **F**: Longitudinal distribution of the proportion of neurological deficits resolved according to the NIHSS (e.g., limb ataxia, motor weakness, dysarthria, etc.) in patients with MGMT-methylated tumors. **G**: Karnofsky Performance Status (KPS) at preoperative (preOP), 8-week, 12-week, and 6-month follow-up, stratified by residual tumor volume in MGMT-methylated tumors. **H**: Proportion of neurological deficits resolved according to the NIHSS (e.g., limb ataxia, motor weakness, dysarthria, etc.) in patients with MGMT-unmethylated tumors (see percentage under E). **I**: Karnofsky Performance Status (KPS) at preOP, 8-week, 12-week, and 6-month follow-up, stratified by residual tumor volume in MGMT-unmethylated tumors (see percentage under E). ns = not significant

### Lower residual tumor volume after re-resection is particularly beneficial for patients with unmethylated *MGMT*

To delineate possible differences between *MGMT* promoter methylated and unmethylated patients, we performed volumetry of T1-CE tumor based on preoperative and early postoperative MRI, which revealed a reduction in median tumor volume from 16.99 to 1.1 ml (*p* < 0.001, Tukey’s multiple comparisons test) in all patients. preopMRI mean tumor volumes (before recurrence surgery) did not differ significantly between *MGMT*-unmethylated and methylated patients (preopMRI 16,91 vs. 17,14, *p* > 0.999, Tukey’s multiple comparisons test) nor did mean volumes on epMRI; 1.01 vs. 1.01 ml, *p* > 0.999, Tukey’s multiple comparisons test), suggesting no inherent bias in CE-RTV by methylation status (Fig. 2A). We noticed a slight switch in methylation status between primary and recurrent glioblastoma cases (62% unmethylated at primary diagnosis vs. 66% at recurrence, Fig. 2B). Based on what is known under the RANO resect study on recurrent glioblastoma, we stratified patients based on residual tumor volume (CE-RTV) into three classes: 0 ml T1-CE tumor = class 1, > 0–1 ml = class 2, and > 1 ml = class 3 T1-CE tumor volume. For patients with methylated (*n* = 58) tumors, differences in post-operative survival between the three CE-RTV classes were noted, albeit statistically non-significant (0 ml 488 days vs. 411 days in > 0–1 ml, *p* = 0.5710 and vs. 378 days in > 1 ml T1-CE, *p* = 0.0701, Log-rank (Mantel-Cox) test, Fig. 2C). However, these differences were more pronounced in patients with unmethylated tumors (*n* = 95, 0 ml = 299 days vs. 288 days in > 0–1 ml, *p* = 0.6837 and 190 days in > 1 ml T1-CE, *p* = 0.0416, Log-rank (Mantel-Cox) test, Fig. 2D). We then compared pooled patients with CE-RTV 0 to 1 ml (RANO classes 1–2) to patients with RANO 3 class resection [16]. Indeed, although both groups derived a significant post-operative survival benefit, *MGMT*-methylated patients showed a rather slim difference (33 days, CE < 1 411 days vs. CE > 1 378 days, *p* = 0.0430. Log-rank (Mantel-Cox) test, Fig. 2E) compared to 98 days in *MGMT*-unmethylated patients (288 days vs. 190 days, *p* = 0.0240. Log-rank (Mantel-Cox) test, Fig. 2F). These differences were less pronounced when examining post-operative progression-free survival (PFS), which is also a known predictor of survival in the context of glioma [19], with similar differences in PFS between 0 ml and > 1 ml CE-RTV groups in methylated (140 days vs. 77 days, *p* = 0.0210, Log-rank (Mantel-Cox) test, Fig. 2I) and unmethylated patients (116 days vs. 53 days, *p* = 0.0122, Log-rank (Mantel-Cox) test, Fig. 2H). Overall, the median survival after surgery was 302 days in all patients (Supplementary Figure S1D), with a longer median postoperative survival in all patients with 0 ml CE-RTV compared to > 1 ml (314 days vs. 226 days, *p* = 0.017, Log-rank (Mantel-Cox) test, Supplementary Figure S1E). Also, there was a survival benefit for patients with methylated vs. non-methylated MGMT promoter (410 days vs. 264 days, *p* = 0.028, Log-rank (Mantel-Cox) test, Supplementary Figure S1F). Refer to Supplementary Tables 1 and 2 for further comparisons between difference RTV classes on MGMT methylated and unmethylated patients.

### Residual tumor volume differences are also *MGMT*-status-specific in patients with recurrent glioblastoma therapy

Given the differences noticed in the influence of CE-RTV on patient survival depending on *MGMT* promoter status, we compared *MGMT*-methylated and unmethylated patients from postoperative therapy, finding no significant difference in any treatment modality between both groups, with a slightly higher proportion of patients receiving TMZ in the methylated group (methylated vs. non-methylated: 28% vs. 17%) and PCV/Lomustine/CCNU in the non-methylated group (methylated vs. non-methylated: 52% vs. 63%, *p* = 0.365, Chi-Squared test, Supplementary Figure S1A, Supplementary Table 1). We compared patients with a CE-RTV of 0 ml, > 0–1 ml, and > 1 ml in terms of post-operative therapy and found no overrepresentations in any of the therapies, neither in patients with methylated (Fig. 3A) nor unmethylated tumors (Fig. 3B). Because the use of alkylating agents (PCV/Lomustine/CCNU) was the most common in this cohort post-operatively, we examined the effect of CE-RTV on post-surgical survival in methylated (*n* = 31) vs. unmethylated patients (*n* = 62) receiving PCV/Lomustine/CCNU. We could also consolidated this effect in PCV/Lomustine/CCNU-treated patients, showing subtle non-significant differences in post-surgical survival in methylated patients with 0 ml CE-RTV (563 days), compared to CE-RTV > 0–1 ml (423 days, *p* = 0.9833 and > 1 ml (470 days, *p* = 0.3741, Log-rank (Mantel-Cox) test, Fig. 3C) which were more pronounced in patients with unmethylated tumors with 0 ml CE-RTV (337 days), compared to CE-RTV > 0–1 ml (254 days, *p* = 0.1041 and > 1 ml (178 days, *p* = 0.012, Log-rank (Mantel-Cox) test. Figure 3D). Further survival analyses revealed no statistically significant differences in patients with post-re-resection irradiation depending on their MGMT-methylation status (*n* = 10, 297 days vs. *n* = 9, 543 days in unmethylated patients, *p* = 0.1559, Log-rank (Mantel-Cox) test, Fig. 3E, with no differences in TMZ treatment in either group, Supplementary Figure S1B, and regardless of CE-RTV, Supplementary Figure S1C). Examining further treatment adjuncts at post-re-resection recurrence, there was no significant difference in survival after second recurrence surgery between MGMT-methylated (*n* = 12, 245 days) and non-methylated tumors (*n* = 11, 279 days, *p* = 0.6381, Log-rank (Mantel-Cox) test. Figure 3F).

### Functional outcomes in *MGMT*-unmethylated and methylated patients after re-recurrence surgery

After showing a specific, more pronounced survival benefit for patients with unmethylated *MGMT* promoter after resection of recurrent glioblastoma, we sought to rule out possible differences in surgical modalities and contextualize the survival data we provide with functional data of neurological deficits and the patient’s general condition. Firstly, all groups were balanced concerning the application of intraoperative MRI (iMRI) across patients with > 1 ml vs. > 0–1 ml and > 1 CE-RTV in patients with methylated and non-methylated *MGMT* protomers; this was also true for awake surgery of resection under intraoperative neuro-monitoring (ioNM, Fig. 4A and B). Secondly, pre-operatively and across the course of 6 months post-operatively, we did not notice any significant differences in median NIHSS of patients between the three CE-RTV groups, neither in methylated nor unmethylated tumors (Fig. 4C and D). We also compared both groups for proportions of patients with relevant deficits at all time points, with NIHSS > 3 set as a stringent threshold, finding no relevant neurological deterioration of patients between pre-operative and discharge NIHSS (19 to 21% in methylated vs. 23 to 26% in unmethylated patients) or overrepresentation of any of the time points or any differences among CE-RTV classes. However, a global deterioration of patients as of 6 weeks after surgery could be noticed, Fig. 4E). We further analyzed the distribution of the nature of these deficits. We found no overrepresentation of deficits that could be regarded as more relevant (motor arm, motor leg, and language) in any of the CE-RTV classes among MGMT-methylated patients (Fig. 4F), which is reflected in the longitudinal Karnofsky Performance Score (KPS) of methylated patients. However, a gradual deterioration over time is noticed in their KPS (Fig. 4G). This was also observed for MGMT-unmethylated patients (Fig. 4H). Notably, at 6 weeks after surgery, patients with 0 ml CE-RTV showed a significantly higher median KPS compared to patients with > 1 ml, while non-significant differences in median KPS between patients with different CE-RTV at 12 weeks or 6 months after surgery were noticed (Fig. 4I), suggesting, in addition to a positive onco-functional situation for patients with 0 CE-RTV and unmethylated tumor an inevitable decline in general condition due to tumor progression over time, similar to patients with methylated tumors.

## Discussion

In this retrospective study, we show that the effect of CE-RTV on survival after re-resection in recurrent glioblastoma is modified by the MGMT promoter status. Patients with unmethylated MGMT promoters derived a markedly greater survival benefit from near-total resection (0–1 ml CE-RTV) compared to those with > 1 ml CE-RTV, whereas the effect was less pronounced in MGMT-methylated tumors. Importantly, maximal resection was not associated with a higher risk of permanent neurological deficits, underscoring the functional safety of maximizing cytoreduction. In addition, patients with unmethylated recurrent glioblastoma and 0 ml RTV showed a significant improvement in KPS at 6 weeks postoperatively compared to their non-methylated counterparts with a higher post-operative tumor burden. Hence, by uniquely addressing both survival and longitudinal functional outcomes this study highlights the particular importance of iMRI-guided maximal safe resection in MGMT-unmethylated recurrent glioblastoma.

Our findings underscore the interplay between molecular tumor characteristics and surgical efficacy in recurrent glioblastoma. We observe not only that patients with a methylated *MGMT* promoter exhibited significantly prolonged survival after re-resection compared to those with an unmethylated promoter, but also that chemosensitivity to alkylating agents appears to partially mitigate the prognostic disadvantage of incomplete resection. Hence, aiming for minimal residual CE volume (ideally ≤ 1 ml) during re-resection may be especially critical in *MGMT*-unmethylated recurrent glioblastoma.

CE-RTV-based stratification thresholds proposed under RANO resect still hold in both *MGMT*-methylated and non-methylated patients under this study [16]. This external validation, along with the similar overall survival of recurrent glioblastoma patients after re-resection surgery (about 11 months), corroborates the existing evidence on volumetric thresholds of resection in recurrent glioblastoma and adds to the generalizability of our findings [1, 2, 16, 28, 33, 34, 37]. It would be justifiable to speculate that patients with MGMT-unmethylated tumors may predominantly drive the observed of the survival benefit associated with *re-resection*, reinforcing the principle of individualized neurosurgical oncology [23].

Because decision-making regarding re-resection is typically based on the *MGMT* status determined from tissue of the primary tumor resection, *MGMT* status at primary glioblastoma diagnosis was used in our analysis. Indeed, we rarely noticed a switch in *MGMT* promoter status between primary and recurrent tumor tissue (4% of the cases). While this noted to be higher in other studies, *MGMT* promoter status is usually regarded to be conserved between primary and recurrent glioblastoma. This stability enables reliable patient identification and stratification before re-resection and may render routine repeat MGMT promoter analyses on tissue sampled at recurrence redundant. Nevertheless, cases of MGMT promoter change have been described, underscoring that repeat testing may be considered in selected clinical scenarios where therapeutic consequences are anticipated [4, 26]. At the same time, emerging intraoperative molecular readouts for somatic variations and epigenomics, including the *MGMT* status, could aid real-time decision-making to tailor resections around residual tumor with oncologically meaningful, achievable resection thresholds in functionally critical regions [7, 8, 27], but equally justify more aggressive surgical goals when re-resection is functionally safe. Although these findings highlight the relevance of maximal safe cytoreduction in MGMT-unmethylated recurrent glioblastoma - they remain exploratory and should not dominate therapeutic decision-making.

Despite significant survival differences driven by CE-RTV, post-re-resection therapy regimens were evenly distributed between *MGMT* groups and CE-RTV classes. Neither the use of alkylating agents (e.g., PCV or CCNU) nor targeted therapies showed significant overrepresentation in CE-RTV classed or *MGMT* status, enforcing the role of surgery in observed differences in CE-RTV-driven prolonged survival. Even in patients receiving PCV/lomustine-based therapies—the most frequently applied regimen in this cohort—observed a more pronounced benefit of lower residual tumor volume (CE-RTV) in patients with an unmethylated *MGMT* promoter, whereas methylated patients showed no explicit CE-RTV-survival dependency. Because the efficacy of lomustine is reported to be restricted to patients with *MGMT* promoter methylated tumors [21, 36, 41], this observation rather reflects retained chemosensitivity in *MGMT*-methylated patients that compensates for limited residual disease and does not necessarily evaluate the impact of PCV/lomustine-based regimens in prolonging post-re-resection survival of recurrent glioblastoma patients. In the small and highly selected subgroup of patients undergoing a second repeat resection (re-re-resection) for further recurrence, we observed a non-significant but discernible survival difference between MGMT-methylated and unmethylated tumors. Given the limited sample size and the inherent selection bias of this cohort, these findings should be interpreted with caution [6, 43].

An essential strength of this study is its annotation with longitudinal functional data, including NIHSS scores up to 6 months after re-resection surgery. Perioperative or delayed neurological deficits were not significantly associated with more aggressive resections in either the *MGMT*-methylated nor the unmethylated group and remained in line with what is reported in earlier studies on recurrent glioma surgery [5, 14, 28, 30, 43]. NIHSS scores and proportions of patients with relevant neurological impairments remained stable across all CE-RTV classes and time points. While transient worsening in neurological function was observed in both groups, a slight functional benefit emerged for patients with 0 ml CE-RTV compared to those with CE-RTV > 1 ml in the *MGMT*-unmethylated group at an intermodal interval after re-resection, rendering maximal cytoreduction not only feasible and justified but also preserving of functional independence, particularly in *MGMT*-unmethylated patients. Ultimately, KPS declined in both *MGMT* groups over time, reflecting disease progression rather than surgical morbidity.

Taken together, our findings advocate for a more tailored surgical approach in the treatment of recurrent glioblastoma. For *MGMT*-unmethylated patients, maximal cytoreduction appears essential to improving survival and should be pursued whenever functionally safe. In contrast, although patients with *MGMT*-methylated tumors demonstrate a substantial baseline prognostic advantage over those with *MGMT*-unmethylated tumors - presumably reflecting different tumor chemosensitivity–the comparatively less pronounced incremental survival benefit observed beyond minimal residual tumor volumes (> 0–1 ml) in the methylated subgroup does not undermine the overall value of re-resection or cytoreduction. Rather, these findings suggest that molecular biomarkers such as MGMT promoter methylation may help individualize surgical resection targets in recurrent glioblastoma, enabling optimization of oncologic benefit with functional preservation, particularly in highly eloquent brain regions [9]. Our findings emphasize the value of molecular genetics as a further adjunct to iMRI, mapping techniques, and refined volumetric thresholds to achieve onco-functional balance in patients with recurrent glioblastoma.

Several real-world factors may limit the feasibility of maximal safe resection in recurrent glioblastoma and should be considered when interpreting these findings. Tumors involving highly eloquent regions often constrain the extent of resection due to the risk of permanent neurological deficits. In the present cohort, awake craniotomy was employed in only 5% of cases and intraoperative neuromonitoring in 16%, reflecting both case selection and practical constraints. Although advanced adjuncts such as intraoperative MRI and neuromonitoring were available at our center, these technologies are not universally accessible, potentially limiting the generalizability of aggressive cytoreductive strategies across institutions.

Limitations of this study include its retrospective nature and the heterogeneity of post-re-resection treatments. Moreover, the relatively limited sample size in specific subgroups (e.g., those receiving re-irradiation or immunotherapy) could undermine the generalizability of treatment-specific survival conclusions, which are beyond the scope of the study. Notably, the prognostic interaction between *MGMT* promoter methylation status and CE-RTV remained clearly observable despite the limited cohort size. Even if the homogeneity of the cohort due to the single-center nature and the almost standardized intraoperative MRI-guided resection could arguably mean a high level of internal consistency, prospective multicenter studies could help corroborate these observations in the future, allowing for a sample size that affords analysis of subgroups with homogenous adjuvant therapies. With prospective studies currently underway (NCT06273176), this effect would be expected to be confirmed in due course. Further studies could also provide a detailed analysis of the impact of surgery on quality of life and neurocognitive performance in patients with recurrent glioblastoma, particularly in patients with impaired function in the peritumoral cortex. This factor has been associated with reduced survival [18]. Quantitative MGMT promoter methylation levels were not analyzed as a continuous variable, as our primary objective was to evaluate MGMT promoter status as a binary, preoperatively available biomarker to inform surgical decision-making at recurrence. Nevertheless, we acknowledge that incorporating quantitative methylation levels may further refine risk stratification and surgical tailoring, and we therefore recognize this as a limitation of the present study and an important avenue for future research.

## Conclusion

This study demonstrates that the prognostic impact of residual tumor volume after re-resection in recurrent glioblastoma is significantly modulated by *MGMT* promoter methylation status. While maximizing resection to 0 ml CE-RTV confers a clear survival advantage in patients with unmethylated tumors, this effect is less pronounced in MGMT-methylated patients. Maximizing resection was not associated with increased postoperative morbidity. These findings reinforce the notion of individualized molecularly informed neurosurgical strategies in recurrent glioblastoma.

## Supplementary Information

Below is the link to the electronic supplementary material.

Supplementary Material 1.
Treatment allocation and survival after re-resection in the total cohort. A: Bar graph depicting distribution of post-operative treatments across MGMT-methylated and unmethylated tumors, Chi-Squared test. B: Number of patients receiving re-irradiation with and without TMZ = temozolomide stratified by MGMT status, Chi-Squared test. Distribution of residual tumor volume (CE-RTV) is equivalent to the extent of resection (EOR) among patients undergoing re-irradiation after re-resection, stratified by MGMT methylation, as assessed by the Chi-Squared test. Kaplan–Meier survival of all patients (n=153) after re-resection (median survival 302 days). E: Survival stratified by CE= contrast enhancing CE-RTV in the total cohort, demonstrating significantly prolonged survival for CE-RTV 0 ml and >0-1 ml compared to CE-RTV >1ml (*p* = 0.017 and *p* = 0.011, respectively, Log-rank (Mantel-Cox) test. F: Overall survival comparing MGMT-methylated and unmethylated tumors, showing significantly longer survival in methylated tumors (median 410 vs 264 days; p=0.0028). Log-rank (Mantel-Cox) test

Supplementary Material 2.

## Data Availability

The datasets generated and/or analyzed during the current study are available from the corresponding author on reasonable request.

## Abbreviations

5-ALA: 5-aminolevulinic acid
ANOVA: Analysis of variance
BCNU: Carmustine
CDK4/6: Cyclin-dependent kinase 4/6
CCNU: Lomustine
CE: Contrast-enhancing
CE-RTV: Contrast-enhancing residual tumor volume
CE-T1: Contrast-enhanced T1 sequence
EOR: Extent of resection
epMRI: Early post-operative MRI
FLAIR: Fluid-attenuated inversion recovery
glioblastoma: Glioblastoma
IDH: Isocitrate dehydrogenase
IDHwt: IDH-wildtype
IQR: Interquartile range
ioNM: Intraoperative neuromonitoring
iMRI: Intraoperative MRI
KPS: Karnofsky performance status
MEK: Mitogen-activated protein kinase kinase
MGMT: O6-methylguanine-DNA-methyltransferase
MRI: Magnetic resonance imaging
NIHSS: National institutes of health stroke scale
OS: Overall survival
PCV: Procarbazine, lomustine (CCNU), vincristine
PFS: Progression-free survival
preopMRI: Pre-operative MRI
recurrent glioblastoma: Recurrent glioblastoma
RANO: Response assessment in neuro-oncology
RTV: Residual tumor volume
SD: Standard deviation
TMZ: Temozolomide
VM-26: Teniposide
VP-16: Etoposide
WHO: World health organization
