# Supplementary figures and images for: MGMT promoter methylation modulates the effect of residual tumor on survival after re-resection of recurrent glioblastoma
Source: Acta Neuropathol Commun. 2026 Feb 12;14:47. doi: 10.1186/s40478-026-02234-w (PMC12930920; doi:10.1186/s40478-026-02234-w)

Supplementary Figure S1

A

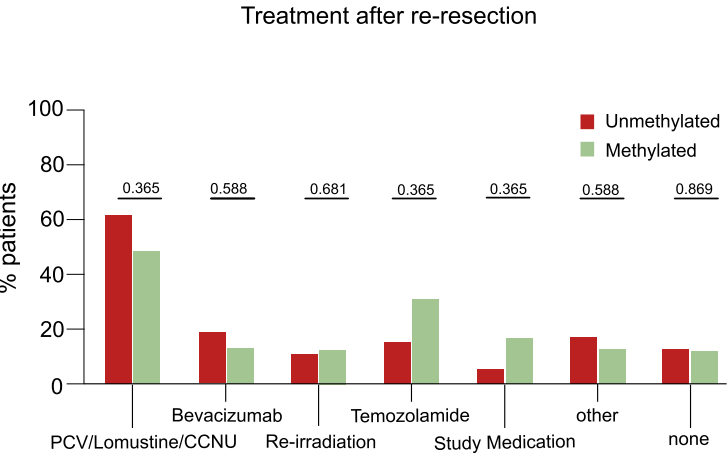

B

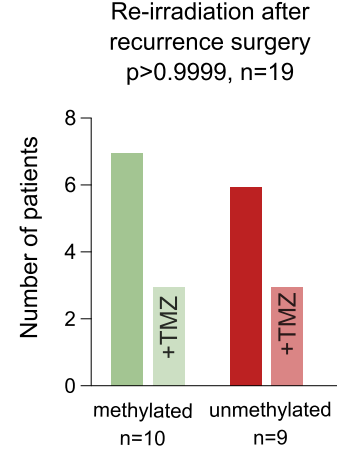

C

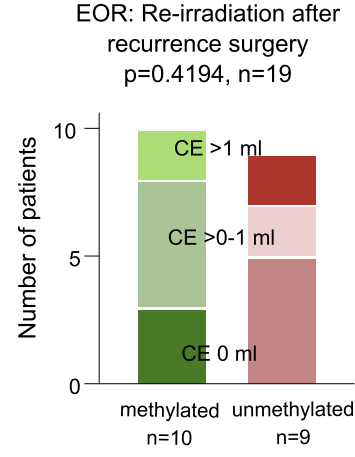

D

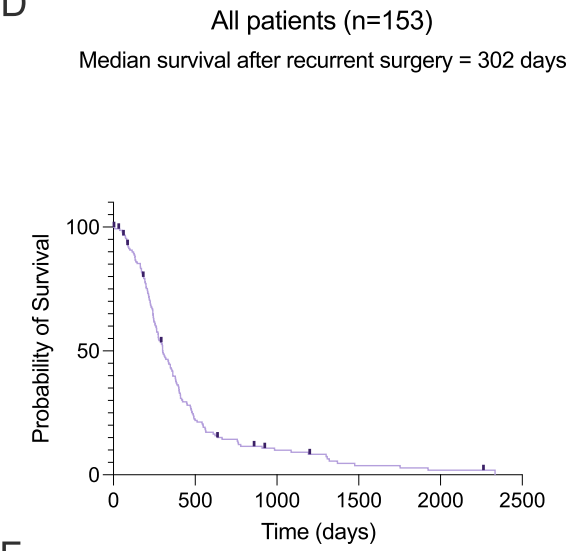

E

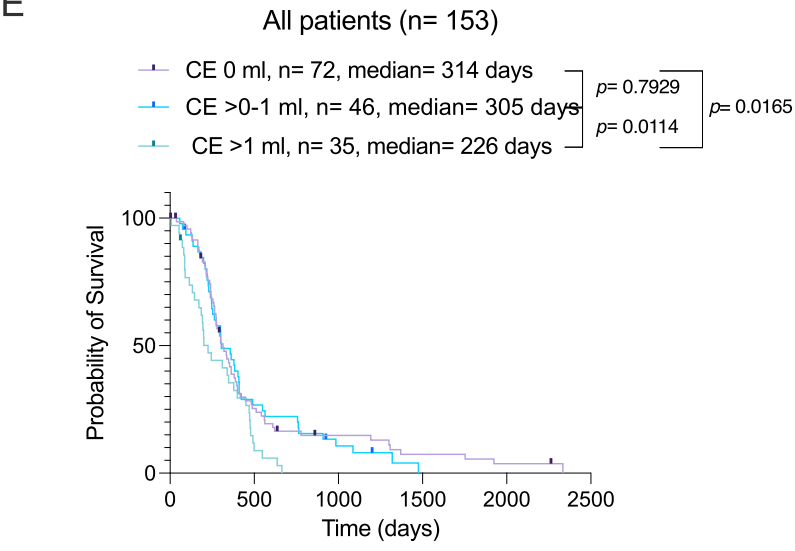

F

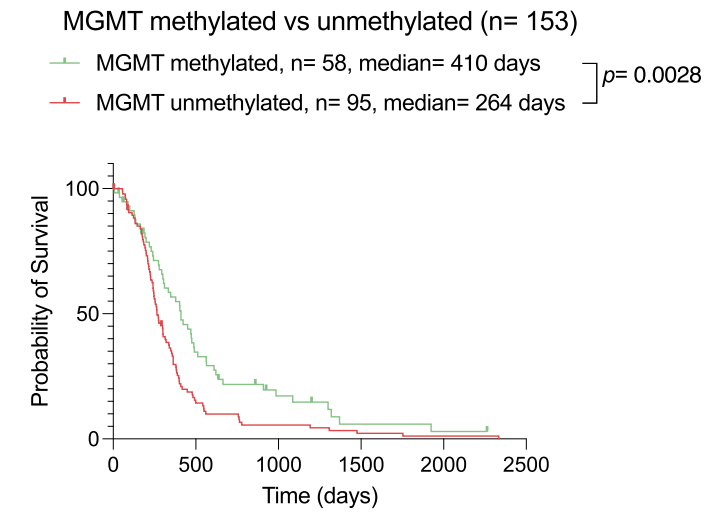

Supplement: Supplementary file 1 — Supplementary Material 1. Treatment allocation and survival after re-resection in the total cohort. A: Bar graph depicting distribution of post-operative treatments across MGMT-methylated and unmethylated tumors, Chi-Squared test. B: Number of patients receiving re-irradiation with and without TMZ = temozolomide stratified by MGMT status, Chi-Squared test. Distribution of residual tumor volume (CE-RTV) is equivalent to the extent of resection (EOR) among patients undergoing re-irradiation after re-resection, stratified by MGMT methylation, as assessed by the Chi-Squared test. Kaplan–Meier survival of all patients (n=153) after re-resection (median survival 302 days). E: Survival stratified by CE= contrast enhancing CE-RTV in the total cohort, demonstrating significantly prolonged survival for CE-RTV 0 ml and >0-1 ml compared to CE-RTV >1ml (p = 0.017 and p = 0.011, respectively, Log-rank (Mantel-Cox) test. F: Overall survival comparing MGMT-methylated and unmethylated tumors, showing significantly longer survival in methylated tumors (median 410 vs 264 days; p=0.0028). Log-rank (Mantel-Cox) test [file 40478_2026_2234_MOESM1_ESM.pdf]
