# Supplementary material for: MGMT promoter methylation modulates the effect of residual tumor on survival after re-resection of recurrent glioblastoma
Source: Acta Neuropathol Commun. 2026 Feb 12;14:47. doi: 10.1186/s40478-026-02234-w (PMC12930920; doi:10.1186/s40478-026-02234-w)
Supplement: Supplementary file 2 — Supplementary Material 2. [file 40478_2026_2234_MOESM2_ESM.docx]

|  | 0 ml (n=24) | >0-1 ml (n=19) | >1 ml (n=15) | p-value |
| --- | --- | --- | --- | --- |
| Gender  Male  Female | 12  12 | 8  11 | 8  7 | 0.790^a^ |
| Age  Mean (SD)  Median (IQR) | 61.2 (10.7)  63.5 (15.0) | 65.9 (7.3)  66.0 (10.5) | 60.0 (10.3)  62.0 (13.5) | 0.186^b^ |
| KPS before surgery  Mean (SD) | 83.8 | 81.6 | 78 | 0.270^b^ |
| Hemisphere  Right hemisphere  Left hemisphere | 11  13 | 10  9 | 10  5 | 0.513^a^ |
| Lobe involvement  Frontal  Parietal  Temporal  Occipital  Insula  Deep | 8  8  13  3  1  0 | 9  6  6  6  1  0 | 6  2  8  3  0  0 | 0.646^a^  0.355^a^  0.280^a^  0.308^a^  0.684^a^  N. A. |
| Treatment  PCV/Lomustine (CCNU)  Bevacizumab  Temozolomide  Study medication^c^  No adjuvant treatment | 13  2  8  2  4 | 11  4  3  3  3 | 7  2  5  0  1 | 0.805^a^  0.485^a^  0.374^a^  0.265^a^  0.647^a^ |
| Re-irradiation | 3 | 5 | 2 | 0.442^a^ |

**Supplementary Table 1: Comparative analysis of the extent of resection classes in patients with methylated-*MGMT* promoter in recurrent glioblastoma**. ^a^Chi-Square-Test. One-way Analysis of Variance (ANOVA). PCV: Procarbazine, lomustine, CCNU, and vincristine. Other agents include carboplatin and hydroxycarbamid. abemaciclib. SD Standard deviation. IQR Inter-quartile Range. *MGMT* Status O6-methylguanine-DNA methyltransferase promoter status.

|  | 0 ml (n=48) | >0-1 ml (n=27) | >1 ml (n=20) | p-value |
| --- | --- | --- | --- | --- |
| Gender  Male  Female | 34  14 | 19  8 | 13  7 | 0.887^a^ |
| Age  Mean (SD)  Median (IQR) | 56.3 (9.9)  56.5 (14.5) | 55.2 (12.7)  55.0 (12.5) | 56.7 (9.9)  58.0 (10.8) | 0.875^b^ |
| KPS before surgery  Mean | 82.5 | 81.15 | 84 | 0.642^b^ |
| Hemisphere  Right hemisphere  Left hemisphere | 21  27 | 12  15 | 7  13 | 0.768^a^ |
| Lobe involvement  Frontal  Parietal  Temporal  Occipital  Insula  Deep | 13  12  25  6  0  0 | 6  7  16  2  0  1 | 8  2  10  3  1  0 | 0.393^a^  0.339^a^  0.780^a^  0.695^a^  0.150^a^  0.280^a^ |
| Treatment  PCV/Lomustine  Bevacizumab  Temozolomide  Study medication^c^  No adjuvant Treatment | 30  9  7  5  6 | 20  5  7  1  3 | 10  4  2  0  3 | 0.237^a^  0.991^a^  0.296^a^  0.221^a^  0.924^a^ |
| Re-irradiation | 5 | 2 | 2 | 0.909^a^ |

**Supplementary Table 2: Comparative analysis of the extent of resection classes in patients with unmethylated *MGMT* in recurrent glioblastoma.**  ^a^Chi-Square-Test. One-way Analysis of Variance (ANOVA). PCV: Procarbazine, lomustine, CCNU, and vincristine. Other agents include carboplatin and hydroxycarbamid. abemaciclib. SD Standard deviation. IQR Inter-quartile Range. *MGMT* Status O6-methylguanine-DNA methyltransferase promoter status.
